# Three-Dimensional (3D) Laparoscopy Versus Two-Dimensional (2D) Laparoscopy: A Single-Surgeon Prospective Randomized Comparative Study

**DOI:** 10.31557/APJCP.2020.21.10.2883

**Published:** 2020-10

**Authors:** Zheng Wang, Jianwei Liang, Jianan Chen, Shiwen Mei, Qian Liu

**Affiliations:** *Department of Colorectal Surgery, National Cancer Center/National Clinical Research Center for Cancer/Cancer Hospital, Chinese Academy of Medical Sciences and Peking Union Medical College, China.*

**Keywords:** Laparoscopy colon resection, colon adenocarcinoma, 3D visualization, operation time

## Abstract

**Background:**

Visual information is crucial for performing laparoscopic surgery. While surgeons lose depth perception and spatial orientation in conventional 2D laparoscopy, the 4^th^ generation 3D system gives a better depth perception.

**Objective:**

In this sstudy, we aimed to investigate the feasibility, safety, and short-term efficacy of 4th generation 3D-HD visualization technology applied in laparoscopic colon cancer surgery.

**Methods:**

One hundred and twenty patients with colon adenocarcinoma were recruited in this study. Patients were randomized on the day of surgery by a random computer-generated allocation list to undergo either a 3D-HD display or 2D-HD imaging system laparoscopic colon cancer surgery. In total, 60 patients underwent laparoscopic colon resection by 3D-HD laparoscope (3D group) and 60 patients underwent 2D-HD laparoscope (2D group). After the insertion of the access ports, both surgical procedures were divided in component tasks, and the execution times were compared. Data analysis was done using SPSS (version 15.0). Quantitative and qualitative variables were compared applying Student t test and Pearson’s chi-square test.

**Results:**

Two groups were homogenous in terms of demographic data. Operation time was significantly shorter for the 3D group than for the 2D group (123.2±34.2 min vs. 142.2±23.5 min, P=0.018). There was no statistically significant difference between two groups in terms of intraoperative blood loss, the number of retrieved lymph nodes, postoperative recovery, and postoperative complications (P>0.05).

**Conclusion:**

The 4^th^ generation 3D-HD vision system reduced the operating time compared to 2D-HD vision system. It seems that use of the 3D-HD technology can significantly enhance the possibility of achieving better intraoperative results.

## Introduction

In 1991, Jacobs et al.,(1991) reported the initial experience of 20 cases of laparoscopic colectomy and proved its technical feasibility. After nearly 20 years of development, laparoscopic surgery for colon cancer has emerged as the standard procedure compared to open surgery bringing out similar oncologic outcomes and superior perioperative results, including earlier return of bowel function, decreased postoperative pain, decreased length of stay, and decreased morbidity (Veldkamp et al., 2005).

However, laparoscopic surgery is more challenging compared with open surgery, in part because surgeons must operate in a 3D space through a two-dimensional projection on a monitor, which does not allow perception of the operative field as in open surgery. Even if the experienced surgeon is able over time to regain some vision of depth. To resolve this problem, 3D imaging for laparoscopic surgery was developed. 

Owing to the limited data regarding the feasibility and safety of 3D visualization technology applied in laparoscopic colon cancer resection, the results on the efficacy of 3D visualization are controversial. In order to analyze the effect of 3D visualization on operative performance, we aimed to highlight the differences between the 4^th^ generation 3D view (3D-HD) and the standard two-dimensional (2D) applied to laparoscopic colon cancer resection in this pilot randomized study. 

## Materials and Methods

*Patients*

The patients recruited for the study had colon adenocarcinoma with full indication for laparoscopic surgery. Between January 2018 and January 2019, a total of 145 patients were diagnosed with colon adenocarcinoma and underwent laparoscopic surgery at the Department of Abdominal Surgical Oncology, Cancer Hospital of the Chinese Academy of Medical Sciences, Peking Union Medical College. Among these 145 patients, twenty five patients who underwent neoadjuvant chemotherapy or had multiple primary colorectal cancer were excluded from the study. To avoid bias, all operations were performed by a single surgeon, experienced in laparoscopy and colorectal cancer surgery, and other two consultant surgeons who acted as the camera operator and assistant. Before starting the study, the surgeon performed four laparoscopic radical resection of sigmoid colon carcinoma in order to be familiarized with the 3D-HD view system. Patients were randomized on the day of surgery. One hundred and twenty operations were performed with either a 3D-HD display or 2D-HD imaging system. In total, 60 patients underwent laparoscopic surgery by the 3D imaging system (3D HD Vision System, 3D group) and 60 patients underwent colon surgery by the 2D imaging system (2D group). The high definition resolution 2D system provides image with similar resolution compared to the 3D system. The 3D view is achieved with the help of a 3D-HD screen and with the use of polarized glasses. The glasses are filtered; each lens only lets one direction of light pass through the eye, thus maintaining two perspectives of the image and giving a tridimensional vision. In the 3D group, there were 40 sigmoid colon resections, 18 right hemicolectomies, and 2 left hemicolectomies. In the 2D group, there were 45 sigmoid colon resections, 12 right hemicolectomies, and 3 left hemicolectomies. 

All patients were informed about the type of visualization (2D or 3D) and full consent were obtained from each of them. The IRB at our hospital approved the study without any ethical concerns.

All the patients routinely were underwent colonoscopy before surgery to identify the disease region and the pathologic type. All patients were diagnosed with colon adenocarcinoma after pathologic examination. The preoperative routine chest x-ray, abdominal ultrasound, and abdominal and pelvic CT examination showed no pulmonary, hepatic, or other distant metastases. All procedures were performed according to the same surgical and oncological principles. The patients in the 3D and 2D groups received similar preoperative assessments and postoperative management.

The following aspects were recorded and compared between 3D and 2D groups: age, sex ratio, body mass index, ASA score, history of abdominal surgery, operation time, intraoperative blood loss, tumor size, T stage, N stage, differentiation of the tumor, specimen length, number of lymph node dissections, surgical procedure, time to first flatus, time to restart of oral diet, time to ambulation, length of hospital stay after operation, and postoperative complications. The specimens were fixed unpinned, examined for margin clearance, and staged according to the seventh edition of the American Joint Committee on Cancer (AJCC) manual. The follow-up time was 30 days after operation.

*Surgical procedures*

All patients were given a mechanical bowel preparation. All patients had urinary catheter and a nasogastric tube. Patients were placed in a supine position with legs apart for a right or left lesion, and patients with sigmoid colon cancer were put in the low Lloyd-Davies. All patients underwent general anesthesia. The same oncologic principles were followed in both groups, i.e., adequate resection margins, en bloc vascular resection and lymphadenectomy, and minimal intraoperative manipulation of the tumor. Surgical procedures included laparoscopic bowel mobilization and blood vessels division, with the specimen being removed through a small skin incision. During right and left hemicolectomy, an extracorporeal end-to-end stapled anastomosis was performed, while during sigmoidectomy the anastomosis was performed by laparoscopic transanal intracorporeal stapled technique. All resections were performed with curative intent. 

*Statistical analysis*

Statistical analysis was performed using SPSS (version 15.0). Results were given as percentages, mean, and standard deviations, or median and ranges. Quantitative and qualitative variables were compared by applying Student t test and Pearson’s chi-square test, respectively. P-value of less than 0.05 was considered statistically significant. 

## Results

The characteristics of the 120 patients are summarized in Table 1.The mean age of 3D group was 58.6±10.4 years (ranging from 25 to74 years), and the mean age of 2D group was 57.8±12.2 years (ranging from 24 to78 years). Patients’ age, sex ratio, the body mass index, and surgical risks were assessed according to the American Society of Anesthesiologists (ASA). Both groups were similar in terms of history of abdominal surgery. 

The operation time was significantly shorter for the 3D group than for the 2D group (123.2±34.2 min vs. 142.2±23.5 min, P=0.018). The intraoperative blood loss was not significantly higher for the 3D group than for the 2D group (38.8±13.4 ml vs. 42.5±10.4 ml, P=0.823). The mean number of nodes resected with 3D group was 19.6±5.4 compared to 18.4±7.6 with 2D group, indicating a significant difference (P=0.865). Tumor size, T stage, N stage, tumor node metastasis staging, and differentiation of the tumor were similar between the two groups. These results are shown in Table 2. 

The time to first flatus, time to restart of oral diet, time to ambulation, and postoperative hospital stay for the 3D group were all similar between groups. Regarding the incidence of postoperative complications, no significant difference was seen between the two groups (6.7% vs. 8.3%, P=0.615). No conversion to open surgery was recorded for either group. There was not reoperation in the 3D group. Reoperation was necessary in one patient in 2D group due to small bowel obstruction. Other postoperative complications were treated conservatively. None of the complications could be assigned specifically to 3D visualization. There was no postoperative death in each group in 30 days. These results are shown in Table3.

**Table 1:** Patient Characteristics of the 3D Group and 2D Group

	3D group (n=60)	2D group (n=60)	P
Age (years)	58.6±10.4	57.8±12.2	0.815
Gender (male / female)	32/28	35/25	0.265
BMI (kg/m^2^)	26.4±3.6	25.5±3.5	0.786
ASA score			0.631
1	4 (6.6%)	7 (11.7%)	
2	45 (75.0%)	41 (68.3%)	
3	11 (18.3%)	12 (20.0%)	
Previous abdominal surgery	2	3	0.521

**Table 2:** Surgical Outcomes of 3D Group and 2D Group

	3D group (n=60)	2D group (n=60)	P
Operation time (min)	123.2±34.2	142.2±23.5	0.018
Intraoperative blood loss (ml)	38.8±13.4	42.5±10.4	0.823
Tumor size (cm)	4.2±1.5	3.8±1.8	0.769
T stage			0.581
pT1	2	4	
pT2	26	32	
pT3	23	20	
pT4	9	4	
N stage			0.749
pN0	15	20	
pN1	35	33	
pN2	10	7	
Stage			0.475
I	14	18	
II	20	22	
III	26	20	
Differentiation			0.793
Well	12	20	
Moderately	27	25	
Poor	19	13	
Mucinous	2	2	
Specimen length (cm)	25.6±3.5	26.5±7.3	0.823
No. of retrieced lymph nodes	19.6±5.4	18.4±7.6	0.889
Surgical procedure			0.425
Right hemicolectomy	18	12	
Left hemicolectomies	2	3	
sigmoid colon resection	40	45	

**Table 3:** Postoperative Recovery and Complications

	3D group (n=60)	2D group (n=60)	P
Time to first passing flatus (days)	2.8±1.3	2.9±2.1	0.765
Time to diet recovery (days)	4.8±1.3	4.9±2.1	0.862
Time to ground activities (days)	3.0±1.2	3.3±1.6	0.754
Postoperative hospital stay (days)	7.6±2.5	7.8±3.2	0.835
Postoperative complications	4 (6.7%)	5 (8.3%)	0.615
Infection of abdominal incision	2	2	
Anastomotic leak	0	0	
Abdominal abscess	0	0	
Intestinal obstruction	1	1	
Urinary retention	0	0	
Urinary tract infection	0	0	
Paralytic ileus	0	0	
Postoperative bleeding	1	2	
Wound dehiscence	0	0	
Deep vein thrombosis	0	0	

## Discussion

Visual information is crucial for performing laparoscopic surgery. Unfortunately, conventional laparoscopy is limited by a 2D vision that does not allow perception of the operative field as in open surgery (Taffinder et al., 1999; Wilhelm et al., 2014). Therefore, surgeons lose depth perception and spatial orientation, and thus experience a higher visual and cognitive load (Kong et al., 2010; Lusch et al., 2014; Smith et al., 2014). For this reason and through the popularity of laparoscopy, besides increasing the resolution of applied camera systems, 3D reproduction of the operative field is improving (van Bergen et al., 2000). 3D display system was introduced in the early 90s, making laparoscopic interventions safer and faster (Hubber et al., 2003; Wagner et al., 2012). However, 3D technology is still not popular at hospitals partly because due to its side effects, a degraded viewing condition from poor image resolution, the requirement to wear uncomfortable eyewear, and the system’s high cost compared with standard 2D equipment (Cicione et al., 2013; Kunert et al., 2013; Alaraimi et al., 2014; Sahu et al., 2014; Wilhelm et al., 2014).

Nowadays, the 4th generation 3D techniques have been improved in comparison to the first generation of 3D vision system introduced in the 90s and can be even replaced by the classic bi-dimensional view (Zobel, 1993; Mueller et al., 1999; Bove et al., 2015). The 4th generation 3D system uses more ergonomic glasses and an innovated technology which gives a better depth perception that cannot be achieved with traditional 2D systems, without any complaints of visual strains (Kong et al., 2010). This depth perception and hand eye coordination were excellent with 3D imaging system in this study, leading to accurate and swift dissection as well as better intra-corporeal knotting to achieve blood vessels division and suture without compromising the safety and operative time.

Up to now, just a few studies on 3D laparoscopic surgery have been done reporting no definite conclusion about its utility. Therefore, this study was designed to assess the feasibility and safety of the 4th generation 3D visualization technology applied in laparoscopic colon cancer resection, and the 4th generation 3D visualization was found to reduce errors and speed the completion of laparoscopic tasks. Our study showed the superiority of the 3D visualization in terms of operative time, resulting in faster surgery. Previous studies reported better results with 3D laparoscopic technique than with the 2D system both in surgical training exercises and different surgical procedures. Practices like linear cutting and suturing, curved cutting and suturing, tubular suturing, and dorsal vein complex suturing simulation were compared between these two techniques, suggesting higher efficacy of 3D in laparoscopy (Peitgen et al., 1996; Badani et al., 2005; Patel et al., 2007).

Transition from the 2D to 3D vision system requires an initial period of adaptation. Once adaptation to 3D view is reached, a more realistic visualization of the surgical field will allow greater speed and precision in the movement of the surgical instrument. Although blood loss was not significantly different between the two groups, the easy identification of small vessels using the 3D vision may reduce blood loss. We believe that this difference can be approved in studies with larger sample size. The 3D vision may offer significant advantages in teaching laparoscopic skills to inexperienced individuals(Votanopoulos et al., 2008). The benefits of 3D system can be improved operative times, shortened learning curves, and greater surgeon comfort. These benefits might allow an inexperienced laparoscopic surgeon to become an expert in laparoscopic surgery faster.

One of the strength of this study was that the comparison between the 2D and 3D surgical procedures was performed by a single surgeon making it more reliable and avoiding any possible bias. Despite this fact, the extensive experience of the surgeon might have influenced the results and complication rates in our study.

In conclusion, our study evaluated the efficacy of 4th generation 3D-HD vision system in laparoscopic colon cancer resection. It was found that3D visualization reduced the operating time compared to high definition 2D. Further large studies, preferably prospective randomized control trials, are required to confirm this finding. Laparoscopic surgeons can benefit from a 3D visualization due to decreased operative time and consequently real clinical improvements. We conclude that the 4th generation 3D laparoscopy may play an important role in the treatment of colon cancer.

## References

[B1] Alaraimi B, El Bakbak W, Sarker S (2014). A randomized prospective study comparing acquisition of laparoscopic skills in three-dimensional (3D) vs two-dimensional (2D) laparoscopy. World J Surg.

[B2] Badani KK, Bhandari A, Tewari A (2005). Comparison of two-dimensional and three-dimensional suturing: is there a difference in a robotic surgery setting?. J Endourol.

[B3] Bove P, Iacovelli V, Celestino F (2015). 3D vs 2D laparoscopic radical prostatectomy in organ-confined prostate cancer: comparison of operative data and pentafecta rates: a single cohort study. BMC Urol.

[B4] Cicione A, Autorino R, Breda A (2013). Three-dimensional vs standard laparoscopy: comparative assessment using a validated program for laparoscopic urologic skills. Urology.

[B5] Hubber JW, Taffinder N, Russell RC (2003). The effects of different viewing conditions on performance in simulated minimal access surgery. Ergonomics.

[B6] Jacobs M, Verdeja JC, Goldstein HS (1991). Minimally invasive colon resection (laparoscopic colectomy). Surg Laparosc Endosc.

[B7] Kong SH, Oh BM, Yoon H (2010). Comparison of two- and three-dimensional camera systems in laparoscopic performance: a novel 3D system with one camera. Surg Endosc.

[B8] Kunert W, Storz P, Kirschniak A (2013). For 3D laparoscopy: a step toward advanced surgical navigation: how to get maximum benefit from 3D vision. Surg Endosc.

[B9] Lusch A, Bucur PL, Menhadji AD (2014). Evaluation of the impact of three-dimensional vision on laparoscopic performance. J Endourol.

[B10] Mueller MD, Camartin C, Dreher E (1999). Three-dimensional laparoscopy Gadget or progress? A randomized trial on the efficacy of three-dimensional laparoscopy. Surg Endosc.

[B11] Patel HR, Ribal MJ, Arya M (2007). Is it worth revisiting laparoscopic three-dimensional visualization? A validated assessment. Urology.

[B12] Peitgen K, Walz MV, Walz MV (1996). A prospective randomized experimental evaluation of three-dimensional imaging in laparoscopy. Gastrointest Endosc.

[B13] Pietrabissa A, Scarcello E, Carobbi A (1994). Three-dimensional versus two-dimensional video system for the trained endoscopic surgeon and the beginner. Endosc Surg Allied Technol.

[B14] Sahu D, Mathew MJ, Reddy PK (2014). 3D laparoscopy - help or hype; initial experience of a tertiary health centre. J Clin Diagn Res.

[B15] Smith R, Schwab K, Day A (2014). Effect of passive polarizing three-dimensional displays on surgical performance for experienced laparoscopic surgeons. Br J Surg.

[B16] Taffinder N, Smith SG, Huber J (1999). The effect of a second-generation 3D endoscope on the laparoscopic precision of novices and experienced surgeons. Surg Endosc.

[B17] van Bergen P, Kunert W, Buess GF (2000). The effect of high-definition imaging on surgical task efficiency in minimally invasive surgery: an experimental comparison between three-dimensional imaging and direct vision through a stereoscopic TEM rectoscope. Surg Endosc.

[B18] Veldkamp R, Kuhry E, Hop WC (2005). Laparoscopic surgery versus open surgery for colon cancer: short-term outcomes of a randomised trial. Lancet Oncol.

[B19] Votanopoulos K, Brunicardi FC, Thornby J (2008). Impact of three-dimensional vision in laparoscopic training. World J Surg.

[B20] Wagner OJ, Hagen M, Kurmann A (2012). Three-dimensional vision enhances task performance independently of the surgical method. Surg Endosc.

[B21] Wilhelm D, Reiser S, Kohn N (2014). Comparative evaluation of HD 2D/3D laparoscopic monitors and benchmarking to a theoretically ideal 3D pseudodisplay: even well-experienced laparoscopists perform better with 3D. Surg Endosc.

[B22] Zobel J (1993). Basics of three-dimensional endoscopic vision. Endosc Surg Allied Technol.

